# The Role of High-intensity and High-impact Exercises in Improving Bone Health in Postmenopausal Women: A Systematic Review

**DOI:** 10.7759/cureus.34644

**Published:** 2023-02-05

**Authors:** Sara Manaye, Kaaviya Cheran, Chinmayee Murthy, Elisa A Bornemann, Hari Krishna Kamma, Mohammad Alabbas, Mohammad Elashahab, Naushad Abid, Ana P Arcia Franchini

**Affiliations:** 1 Department of Internal Medicine, California Institute of Behavioral Neurosciences & Psychology, Fairfield, USA; 2 Department of General Medicine, California Institute of Behavioral Neurosciences & Psychology, Fairfield, USA; 3 Department of Neurology, California Institute of Behavioral Neurosciences & Psychology, Fairfield, USA; 4 Department of Psychiatry, California Institute of Behavioral Neurosciences & Psychology, Fairfield, USA; 5 Department of Internal Medicine, University of Debrecen, Debrecen, HUN; 6 Department of Radiology, California Institute of Behavioral Neurosciences & Psychology, Fairfield, USA; 7 Department of Internal Medicine, King Faisal University, Alahsa, SAU; 8 Department of Research, California Institute of Behavioral Neurosciences & Psychology, Fairfield, USA

**Keywords:** postmenopausal women, osteoporosis, bone mineral density, high intensity interval training, high impact exercise, high intensity exercise

## Abstract

Postmenopausal osteoporosis is a chronic condition with decreased bone mass and altered bone structure, leading to a greater risk of fractures among older women. Exercise has been proposed as a potentially effective non-pharmacological method to prevent this condition. In this systematic review, we investigate the effects and safety of high-impact and high-intensity exercises in improving bone density at popular sites of fragility fractures, namely, the hip and spine. This review also highlights the mechanism of these exercises in improving bone density and other aspects of bone health in postmenopausal women.

This study is done adhering to the Preferred Reporting Items for Systematic Reviews and Meta-Analyses (PRISMA) guidelines. After applying the eligibility criteria, we selected 10 articles from PubMed and Google Scholar to be included in our study.

Based on the findings from the studies, we established that high-intensity and high-impact exercises are effective in improving, or at the very least maintaining, bone density in the lumbar spine and femur in postmenopausal women. An exercise protocol including high-intensity resistance exercises and high-impact training is shown to be most effective in improving bone density and other parameters of bone health. These exercises were found to be safe in older women, however, careful supervision is recommended. All limitations considered, high-intensity and high-impact exercises are an effective strategy to enhance bone density, and potentially reduce the burden of fragility as well as compression fractures in postmenopausal women.

## Introduction and background

Osteoporosis is a systemic disease characterized by a reduction in bone mass density and micro-architectural changes in bone structure, leading to an increased risk of fractures [1]. Popularly known as the silent disease, osteoporosis is usually only diagnosed following fractures that occur with minimal or no trauma [2]. While 200 million people around the world have osteoporosis, it is more prevalent among postmenopausal women [3,4]. 

Postmenopausal osteoporosis is a chronic condition attributed to aging and estrogen deficiency in older women [1]. Osteoporotic fractures have an enormous impact on aging women's health and the economy of society and nation [5]. This can also adversely affect the quality of life of women due to chronic pain and functional disability [4,6]. The most commonly encountered sites of osteoporotic fractures in this population are the hip and spine [7]. Nonetheless, there are preventive measures that can hinder the progression of osteoporosis, including calcium and vitamin D supplementation, avoidance of excessive alcohol intake and smoking, and exercises providing ground and joint reaction force [8]. It is also important to maintain normal bone density as a preventive measure against osteoporotic fractures during the premenopausal years, as the risk of fractures increases with age [9]. 

There are existing data highlighting the advantages of exercise in improving bone metabolism and bone density, making physical exercise a potentially effective non-pharmacological treatment for postmenopausal osteoporosis [7,10]. The effects of exercise in improving bone health have been extensively researched, however, since not all types of exercise are beneficial in enhancing bone density, the results of these studies remain inconclusive [11]. Frost's Mechanostat theory suggests that exercises that produce a high mechanical loading can improve various bone properties, including its mass, geometry, and strength [7]. Notably, bone tissue reacts better to exercises of high intensity and magnitude, but guidelines recommend only low to moderate intensity due to the anticipated risk of falls, fractures, and concerns over the safety of high-intensity exercises [12]. Moreover, to elevate bone density, bone tissue should be subjected to physical loads above what is normally experienced during daily physical activities [13]. The high mechanical loads lead to the thickening of cortical and trabecular bone, effectively strengthening the bone structure [14]. Exercises that target the most common sites of fractures need to be advocated via gravitational loading or muscle contraction, as bone adaptions are site-specific [15].

Overall, exercise has been prescribed as a constructive method for enhancing bone strength and decreasing fracture risk; however, optimal exercise prescription and compliance are vital [15]. Most studies examine bone mineral density (BMD) as an indicator of bone health, albeit, other parameters like bone structure and geometry may provide more valuable information [16].

This systematic review’s objective is to establish the effects of high-intensity and high-impact exercises in improving various aspects of bone health in postmenopausal women. The observation of our review emphasizes the effect of various land and aquatic exercises on the BMD changes in the lumbar spine (LS) and femur. Moreover, the safety of these exercises on postmenopausal women is a matter of concern and has been discussed in this systematic review. 

## Review

Methods

**Figure 1:**
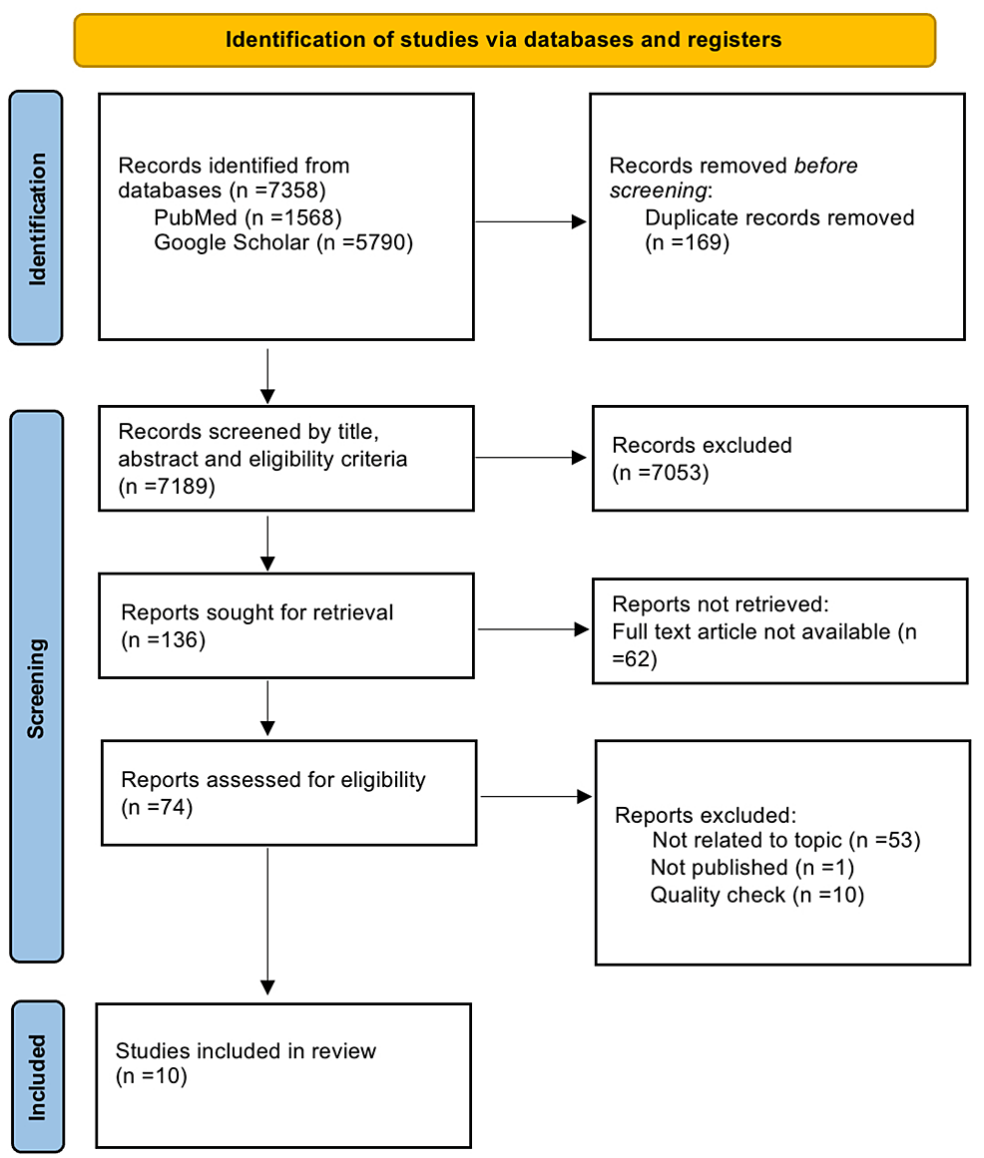
PRISMA Flow Diagram PRISMA - Preferred Reporting Items for Systematic Reviews and Meta-Analysis

This systematic review was done following the Preferred Reporting Items for Systematic Reviews and Meta-Analyses (PRISMA) 2020 guidelines [17]. A complete PRISMA flow diagram is shown in Figure 1.

Information Sources

An extensive search was conducted for studies that dealt with the effects of high-intensity and high-impact exercises on different aspects of bone health in postmenopausal women. This research included randomized control trials (RCT), systematic reviews (SR), and meta-analyses (MA) done in the last 10 years (2012-2022). The Preferred Reporting Items for Systematic Reviews and Meta-Analysis (PRISMA) 2020 Guidelines were followed in this study. We used major literature databases, including PubMed as the primary source and Google Scholar as the secondary source, to find relevant studies.

Search Strategy

We used the following keywords for our literature search: high-intensity exercise, high-impact exercise, high-intensity interval training, bone mineral density, osteoporosis, and postmenopausal women. Relevant Medical Subject Headings (MeSH) were selected for the keywords, and the following Mesh approach was created using Boolean AND and OR for PubMed search:

High intensity exercise OR High intensity interval training OR High impact exercise OR ["High intensity Interval Training/epidemiology"[Majr] OR "High intensity Interval Training/methods"[Majr] OR "High intensity Interval Training/pharmacology"[Majr] OR "High intensity Interval Training/standards"[Majr] OR "High intensity Interval Training/statistics and numerical data"[Majr] OR "High intensity Interval Training/therapeutic use"[Majr] ] AND Postmenopausal osteoporosis OR Postmenopausal osteopenia OR Postmenopausal bone loss OR [ "Osteoporosis, Postmenopausal/diagnosis"[Majr] OR "Osteoporosis, Postmenopausal/drug therapy"[Majr] OR "Osteoporosis, Postmenopausal/epidemiology"[Majr] OR "Osteoporosis, Postmenopausal/pathology"[Majr] OR "Osteoporosis, Postmenopausal/physiology"[Majr] OR "Osteoporosis, Postmenopausal/physiopathology"[Majr] OR "Osteoporosis, Postmenopausal/prevention and control"[Majr] OR "Osteoporosis, Postmenopausal/statistics and numerical data"[Majr] OR "Osteoporosis, Postmenopausal/therapy"[Majr]

The search strategy used to find relevant articles is depicted in Table 1.

**Table 1:** Search strategy MeSH: medical subject headings

Database	Search strategy	Results
PubMed	MeSH strategy as above	1363
PubMed	High intensity exercise OR High impact exercise AND Bone mineral density OR Osteoporosis AND Postmenopausal women	205
Google Scholar	High intensity exercise OR High intensity interval training OR High impact exercise AND Bone mineral density OR Osteoporosis AND Postmenopausal women	5790

Eligibility Criteria

Only articles written in English, published since 2012, and available as full-text articles were included. Postmenopausal women were chosen, and studies that dealt with the effects of high-intensity and high-impact exercises on various bone health parameters were selected for screening. Animal studies and studies that focused on men and premenopausal women were omitted.

Data Collection

Articles selected through the search approach were imported to Endnote (Clarivate, Philadelphia, PA), and the duplicates were deleted. The remaining articles were screened by title and abstract individually by two different authors. Full-text articles were collected for the studies that provided the required vital evidence. 

Analysis of the Quality of Studies

Articles selected were screened using quality appraisal tools. The Assessment of Multiple Systematic Reviews (AMSTAR) tool was used to screen the systematic reviews/meta-analyses. The Cochrane bias assessment tool was used to screen the RCTs. 

Results

We found a total of 7358 articles through our initial search in PubMed and Google Scholar; 169 duplicates were detected and deleted using the online version of Endnote. We further excluded 7053 articles by title and abstract screening. One hundred and thirty-six articles were sought for retrieval, of which 74 full-text articles were retrieved and were assessed for eligibility. Through the application of eligibility criteria, the 10 most relevant, moderate- to high-quality articles were chosen to be included in our systematic review. A summary of the clinical studies is tabulated in Table 2.

**Table 2:** Study characteristics, results, and conclusions from the selected studies RCT: randomized control trial; HiRIT: high-intensity resistance and impact training; CG: control group; LS-BMD: lumbar spine-bone mineral density; BMC: bone mineral content; EG: exercise group, FN-BMD: femur neck-bone mineral content; FN: femur neck; LS: lumbar spine; BMD: bone mineral density; SR: systematic review; MA: meta-analysis

Author/year	Study type	Exercise intervention	Sample size (dropouts)	Results and conclusion
Kistler-Fischbacher et al. 2021 [18]	RCT	Supervised HiRIT for 40 minutes on non-consecutive days, twice-weekly for eight months. Low-intensity exercises for 40 minutes on non-consecutive days, twice-weekly for eight months (CG)	HiRIT- 42 (1) CG - 44 (5)	There was a positive correlation between maximum weight lifted and changes in LS-BMD in the HiRIT group. High-intensity training led to significant improvement in the indices of fracture risk
Multanen et al. 2014 [19]	RCT	High-impact, multidirectional, aerobic, and step aerobic jumping exercise programs alternating every two weeks. Supervised group exercise classes lasting 55 minutes three times a week for 12 months	40 (4)	BMC at the femur neck was significantly greater in the exercise group. There were no significant differences in the trochanter or lumbar spine
Watson et al. 2018 [20]	RCT	Supervised HiRIT for 30 minutes, twice weekly for eight months (EG). Home-based low-intensity exercise program for 30 minutes, twice weekly for eight months (CG)	EG - 49 (6) CG - 52 (9)	Effects were superior in the HiRIT group for LS- BMD, FN- BMD, FN cortical thickness, height, and all functional performance measures
Hettchen et al. 2021 [21]	RCT	High-impact weight-bearing/high-intensity/velocity resistance training, three times a week for 13 months (EG). Low-intensity exercise, once a week for 13 months (CG)	EG - 27 (6) CG -27 (7)	LS-BMD was maintained in the EG and decreased in the CG, with a significant difference between the two groups
Aboarrage et al. 2018 [22]	RCT	High-intensity jump based aquatic exercise program, 30-minute sessions, three times a week over 24 weeks	15 (0)	A significant increase in LS, total femur and whole body BMD and functional fitness parameters were observed in the training group
Pinho et al. 2020 [23]	RCT	High-impact exercises and power training, thrice-weekly sessions of 60 minutes on non-consecutive days for 20 weeks	21 (0)	The training group showed significant improvements in LS trabecular bone score, distal tibia trabecular thickness, and trabecular BMD
Moreira et al. 2013 [24]	RCT	High-intensity aquatic exercise sessions lasting for 50 to 60 minutes, three times a week for 24 weeks, in a covered swimming pool	64 (5)	There was an increase in bone formation markers with less considerable rise in bone resorption markers in the exercise group. The femoral trochanter BMD presented a reduction in the CG, whereas no change was observed in the EG
Basat et al. 2013 [25]	RCT	High-impact exercises for 60 minutes, three sessions a week for six months. Strengthening exercises for 60 minutes, three sessions a week for six months	High impact - 14 (2) Strengthening - 14 (3)	There was a significant increase in the BMD at the LS and FN in the high-impact group compared to the strengthening group
Sañudo et al. 2017 [13]	SR	Ten studies were included, and the exercise interventions were categorized as dynamic weight-bearing exercises with progressive resistance strength training; vibration training providing asymmetric impacts, together with high-impact training sessions; and studies that combined more than one of the above exercise interventions	989	Significant changes in LS-BMD, and FN-BMD were found mainly with high-impact exercise and whole-body vibration interventions. Impact exercises combined with other forms of training (vibration or strength training) are effective in preserving BMD
Kistler-Fischbacher et al. 2021 [26]	MA	Exercise intensity was classified as low for 19, moderate for 40, and high for four interventions	EG - 1948 CG- 1582	High-intensity exercise is a more effective stimulus for LS-BMD than low or moderate intensity, but not FN-BMD. A positive relationship between load magnitude and bone response was demonstrated

Discussion

Postmenopausal osteoporosis is an important global health problem, and an effective treatment strategy remains an essential line of research [18]. The effectiveness and safety of high-intensity exercises in increasing bone health in postmenopausal women have only been explored in some trials [18]. In a systematic review, Sañudo et al. reported that the predominant factor affecting BMD is the exercise intensity and type, whereas the duration of the exercise trials had no significance [13]. Kistler-Fischbacher et al. concluded that progressive resistance training of high intensity, along with weight-bearing impact activities, is the best exercise prescription to improve BMD in postmenopausal women [26].

This systematic review aims at determining the efficacy, mechanism, and safety of high-intensity and impact exercises in improving BMD and other indicators of bone metabolism in postmenopausal women. The effects on bone density in the lumbar spine and proximal femur have been explored with land as well as water-based high-intensity exercises. We have included eight RCTs and two systematic reviews/meta-analyses in this review. 

Effects of High-intensity and High-impact Exercises on the Lumbar Spine

In two randomized trials, eight months of high-intensity resistance and impact training (HiRIT) in postmenopausal women showed improved LS-BMD. Both trials increased height and improved kyphosis, indicating a reduced risk of vertebral fractures. Resistance exercises comprising deadlifts, overhead presses, and back squats, with high-impact jump exercises were included in the HiRIT protocol [18,20]. A series of high-intensity weight-bearing, jumping, and resistance exercises lasting 13 months, maintained the LS-BMD in the participants. However, the effects were modest compared to other trials, and this might be due to the unexpected early cessation of the study and the gradual increase in exercise intensity resulted in participants being trained at lower intensities for the first five to six months, and the threshold for bone remodeling not being attained [21]. 

An association between maximal weightlifting and an increase in LS-BMD, as observed in the Medication and Exercise for Osteoporosis (MEDEX-OP) trial, suggests a load-response relationship [18]. In a meta-analysis, high-intensity exercises were shown to positively affect the LS-BMD than low to moderate-intensity exercises [26]. Kistler-Fischbacher et al. used a high cut-off for high-intensity exercises, wherein resistance training at >80% one-repetition maximum (1-RM) in sets of six or fewer repetitions, and impact training, creating a ground reaction force (GRF) of four times the body weight were selected, which led to fewer studies in the high-intensity category [26]. There was also an emphasis on the lack of existing information in the high-intensity category and the requirement for more trials to determine the importance of this intervention [26]. A trial with high-intensity impact exercises with progressive external loading, and strength training with increased internal loading for 20 weeks showed significant improvement in the LS-trabecular bone score, pointing towards improved bone microstructure that is more resistant to fractures [23]. The high impact and muscle compression force produced from high-intensity/impact exercises may slow down the process of bone aging and contribute to better functional capacity in postmenopausal women [23]. 

A noteworthy improvement in the LS-BMD was observed following a high-intensity jump-based aquatic exercise program lasting 24 weeks, highlighting the need for exercises that exert a mechanical stimulus above what the body is adjusted to, in order to stimulate osteogenesis. In this trial, an intensity regarded as maximal was selected by the participants and was monitored by assessment of the perceived exertion [22]. The mechanical stimulus produced by exercise loading leads to cellular activation and complex biochemical reactions in the osteocytes [22]. Another high-intensity aquatic exercise program involving strength training and cardiorespiratory training, monitored using the Borg Scale of perceived exertion, showed no LS-BMD improvement [24]. Basat et al. found increased LS-BMD, improved bone formation, and reduced rate of bone loss following a high-impact, jump rope-based exercise program for six months in postmenopausal women [25]. However, another high-impact jump exercise training, producing acceleration peaks higher than 3.9 g, showed no effect on the LS-BMD [19]. Sañudo et al. reported that studies using high-impact training such as running or jumping, produce high peak forces to the bone, triggering bone formation and contributing to improved LS-BMD [13]. Short, intense, bone-loading exercise sessions with intermittent periods of rest are better known to produce an osteogenic response [13]. 

Role of High-intensity and High-impact Exercises in Altering Femoral BMD and Strength

In the Lifting Intervention for Training Muscle and Osteoporosis Rehabilitation (LIFTMOR) trial, eight months of resistance exercises at >80-85% 1-RM, with high-intensity impact training, resulted in increased femur neck (FN)-BMD and FN cortical thickness in the exercise group, whereas in the eight-month MEDEX-OP trial, similar HiRIT showed improved proximal femur strength [18,20]. Watson et al. highlighted that increased cortical thickness is the dominant factor in improving FN strength and thus helps prevent hip fractures [20]. Studies using high-impact exercises resulted in increased FN-BMD, and these changes were dependent on the loading magnitude [13]. FN-BMD is receptive to high-impact exercises, and a high GRF of at least two times the body weight is required to stimulate an osteogenic effect [26].

In a jump-based, aquatic, high-intensity interval exercise program (HIIAE) lasting 24 weeks, Aboarrage et al. observed a considerable increase in the total femur BMD [22]. As understood, the resistance provided by water in aquatic exercises leads to increased musculoskeletal efforts, contributing to increased bone density [22]. A specific aquatic exercise of high intensity showed increased levels of markers of bone formation and a decrease in the levels of bone resorption markers in the exercise group, leading to maintained femoral trochanter BMD in the exercise group at the end of the study in 24 weeks. This has been attributed to performing high-velocity movements against water resistance, providing stimuli for sufficient bone metabolism [24]. Along with loading exercises, exercises involving strong muscle contractions also contribute to improving bone density [24]. Effectively, high-intensity exercises result in the activation of fast-twitch muscle fibers which is likely to accelerate bone development [22]. 

Multanen et al. reported that 12 months of high-impact, aerobic and step-aerobic, multi-directional, jumping exercise training resulted in a substantial rise in the FN-BMD and lowered the osteoporotic fracture risk in postmenopausal women with mild osteoarthritis [19]. Frequent bone loading, particularly, high-impact loading, is essential to potentiate an osteogenic effect wherein bone tissue alters its mass, structural strength, and architecture [19]. Similarly, another high-impact exercise trial for six months induced high mechanical loading at the femoral neck and effectively increased the FN-BMD [25]. 

Safety in Postmenopausal Women

The safety of high-intensity and high-impact exercises in postmenopausal women has been a matter of concern due to the risk of fragility fractures. [18]. High-intensity exercises are traditionally considered unsafe and are less recommended in postmenopausal women [20]. However, several trials have reported this as a safe and effective intervention if done under supervision and with the right technique [18,20]. After the MEDEX-OP trial, no incidence was reported that suggests high-intensity, high-impact jumping exercise, and resistance training as an unsafe intervention for postmenopausal women [18]. Similarly, the LIFTMOR trial of eight months had no fractures or major injuries reported by the participants, thus making it a safe exercise prescription in postmenopausal women with decreased bone mass [20]. Additionally, no participants in the study by Aboarrage et al. reported any injuries, and the program was identified to be safe for postmenopausal women [22]. In a 12-month high-impact training, exercise-related physical symptoms were reported, however, they were short-lived, and the participants could continue their training without complications [19]. Kistler-Fischbacher et al. did not report any unfavorable effects in the high-intensity exercise group compared to the low-intensity exercise group [26]. As such, a comprehensive exercise program involving stability, functional, and strength training can improve physical performance and parameters of bone strength, leading to decreased risk of falls and consequential fractures amongst postmenopausal women [20]. Notably, a high-intensity exercise prescription is safe and does more good than harm, however, a thorough initial assessment, personalization, and supervision of the exercises are required to ensure patient safety [18].

Limitations

Our review was restricted to studies in the English language, published during and after 2012. The studies included were conducted mostly on healthy women with no other comorbidities; therefore, the results cannot be generalized to all postmenopausal women. The included studies use different scales and methods to measure the intensity/impact of the exercises. The measure of the intensity of the exercises is not uniform throughout the included studies, and there is wide variability between the type and duration of the exercise interventions leading to a lack of a definitive conclusion. Since exercise interventions cannot be blinded from the participants, there is a risk of bias in the results. More randomized trials with large sample sizes and longer periods of high-intensity training, with an exploration into other indices of bone health, including bone morphology and bone turnover markers, are recommended as a future line of research. 

## Conclusions

After carefully reviewing the selected articles, we concluded that high-intensity and high-impact exercises effectively mitigate postmenopausal osteoporosis. Strengthening exercises, jumping exercises, and weight-bearing aerobic exercises that exert a high magnitude loading on the bone and produce strong muscle contractions are shown to improve or maintain the lumbar spine and femur bone density. These exercises include resistance training and impact training at high intensities, wherein high intensity can be a measure of 1-RM, GRF, maximal heart rate, or perceived exertion. Moreover, aquatic exercises producing strong muscular efforts are also effective in stimulating bone formation. It is important that these exercises are incorporated as a lifestyle well in advance to prevent the detrimental effects of postmenopausal osteoporosis and fragility fractures. These exercise trials were concluded to be safe for older women, however, supervision of the correct technique is recommended. Larger studies on high-intensity and high-impact exercises with long-term follow-up on compliance and fracture occurrence are essential queries for future research to answer.
